# Body creams and electric screwdrivers: how counter-stereotypical but trivial tasks trigger gender identity threat

**DOI:** 10.3389/fpsyg.2025.1675146

**Published:** 2025-12-01

**Authors:** Gloria Jiménez-Moya, Ana N. SanMartín, Héctor Carvacho

**Affiliations:** Escuela de Psicología, Falcultad de Ciencias Sociales, Pontificia Universidad Católica de Chile, Santiago, Chile

**Keywords:** gender identity threat, gender roles, gender stereotypes, counter-stereotypical roles, sexism

## Abstract

Traditional gender roles are fixed social norms that dictate what men and women should and should not do. These gender patterns have prejudicial consequences for both men and women. As such, reducing support for and approval of gender roles is a crucial goal in our societies. Exposure to counter-stereotypical role models and interventions showing men and women in cross-gendered roles seems to be an efficient way of reducing the impact of traditional gender norms. However, holding a cross-gendered position might trigger negative backlash in terms of identity. In this work, we test the effect of a common and simple counter-stereotypical choice on the emergence of gender identity threat. Participants (*N* = 493) who were forced to choose a counter-stereotypical product for themselves (a body cream or an electric screwdriver) showed compensatory gender-system defense responses. Men who experienced gender identity threat subsequently expressed higher levels of sexism, whereas women under threat showed greater support for the traditional feminine stereotype. These results suggest that even counter-stereotypical everyday and trivial tasks can trigger a gender identity threat that, in turn, leads to attitudes that justify and perpetuate gender inequality.

## 1 Introduction

The fact that women are able to lead and that men should take care of domestic tasks seems beyond discussion. There is a social agreement that we should overcome traditional gender roles because both men and women can exhibit a wide range of characteristics and are capable of effectively managing different tasks, regardless of their sex. The apparent fall in support for the division of roles according to sex might constitute a means of achieving gender equality (Eagly and Koenig, 2021). However, there are still barriers that prevent people from endorsing the overthrow of gender stereotypes and roles (e.g., IPSOS, 2024; Jiménez-Moya et al., 2022a; Sczesny et al., 2025). We argue that carrying out specific roles that have traditionally been assigned to the other sex might still lead to a perceived threat to gender identity. This threat, in turn, will predict the restoration of gender status and inequality through support for traditional gender attitudes. In this work, we manipulated the perceived threat to gender identity by forcing participants to choose a type of product commonly associated with a specific gender role—traditionally masculine or feminine—and we confirmed that gender identity threat might lead to gender status restoring attitudes.

Traditional gender roles are defined as beliefs regarding the qualities and behaviors that are appropriate for men and women in a society (Eagly, 1987). Primarily, they establish that women are suited to domestic and caregiving matters (Ellemers, 2018). Related dimensions such as beauty, emotional skills, and interpersonal relationships emerge as central to the female stereotype (Eagly and Koenig, 2021), and women are often judged based on these traits (Allan, 2009; Wolf, 2002). Men are expected to manage public roles, such as financial and political subjects. Professional success, autonomy, self-confidence, and manual skills are central to the male stereotype (Kite et al., 2008). According to social role theory, both biological and social differences interact to create this division of labor and roles between men and women. Beliefs about gender roles act through biosocial processes, producing gender-differentiated behaviors, cognitions, and affects (Eagly, 1987). As individuals develop, this division is learnt and reinforced through socialization, direct experiences, and modeling, which convert beliefs about men and women into gender roles (Eagly and Wood, 2012).

### 1.1 Why gender roles are harmful and how they might be transformed

Gender roles have a negative impact on attitudes and beliefs and contribute to perpetuating gender inequality in several ways, such as shaping and sustaining gender stereotypes. Individuals’ beliefs regarding a group’s typical occupational roles predict the stereotype of that group. In other words, beliefs concerning the attributes associated with a group’s typical roles are related to perceptions of the traits of this social category in terms of both agency and communality (Koenig and Eagly, 2014; Steinmetz et al., 2014). This implies that group stereotypes cannot be changed if roles are maintained. Gender roles also affect attitudes and behavior, such as levels of self-reported empathy (Löffler and Greitemeyer, 2021) or choice of academic field (Reuben et al., 2014). According to social role theory, men are underrepresented in communal roles and occupations (e.g., Croft et al., 2015), whereas women are underrepresented in STEM (science, technology, engineering, and mathematics) careers (Liñán et al., 2022; Schmader, 2023). This gap is also related to the fact that gender roles shape financial expectations (e.g., D’Acunto et al., 2021), and that women experience higher levels of burnout related to paid work compared to men (Artz et al., 2022).

It seems logical that the emergence and strengthening of counter-stereotypical role models might have a positive impact on reducing support for gender roles. Showing women in typically masculine roles—such as politics or finance—and men in traditionally feminine roles—such as caregiving or domestic—would serve as evidence of the fact that the gender division of roles is not unalterable (e.g., Olivetti et al., 2020). Longitudinal interventions promoting counter-stereotypical behavior have a positive effect on the goals and behaviors of preadolescents and children, especially those that involve the whole community and include clear encouragement or follow-up actions (Olsson and Martiny, 2018). Furthermore, women who admire counter-stereotypical female role models show higher levels of participation in STEM and other typically male-dominated labor markets (Chhaochharia et al., 2022), and exposure to women in science can reduce negative stereotypes regarding women’s intellectual skills (Buckley et al., 2022).

Counter-stereotypical modeling thus emerges as an efficient way of tackling the endorsement of gender roles and, in turn, of mitigating its effects. However, this does not happen in all cases as counter-stereotypical information about women shows to be less effective at reducing female stereotypes, compared to counter-stereotypical information about men when reducing male stereotypes (Cao and Banaji, 2016; Guerra et al., 2021). Furthermore, embracing counter-stereotypical roles might have negative consequences in terms of identity (Rudman et al., 2007).

### 1.2 Gender identity threat and its consequences

Gender roles define social norms regarding what behaviors, occupations, work, and tasks are (un)suitable for men and women (Eagly and Karau, 2002; Heilman and Parks-Stamm, 2007). As such, there is normative pressure on women to comply with the feminine stereotype and for men to fit the masculine stereotype. What are the consequences of deviating from these socially rooted gender patterns? Deviations from these gender stereotypes are met with penalties (Burke et al., 2024; Berger et al., 2022; Morgenroth et al., 2022). An extensive body of research has shown over the decades that a failure to conform to gender roles leads to severe consequences. Agentic women who are seen as highly qualified to do a given job are also perceived as socially unlikable; communal men are viewed as agreeable but less competent than agentic men (Adamus and Ballová Mikušková, 2024; Raymondie and Steiner, 2022; Rosette et al., 2015; Rudman and Glick, 1999, 2001). Women leaders who have a directive style receive more negative evaluations than those who exhibit a participatory style (Eagly et al., 1992), and are less persuasive when their style is task-oriented rather than people-oriented (Carli et al., 1995). Women in positions of leadership are less liked, especially if they fail (Süssenbach and Carvacho, 2022). Women who prefer not to confront a prejudiced event—congruent with a feminine role—are evaluated more positively by sexist individuals compared to women who confront the situation, displaying a typically masculine behavior (Jiménez-Moya et al., 2022b). In sum, women are more likely to face backlash when behaving in a counter-stereotypical manner (Rudman and Phelan, 2008), and men also suffer negative outcomes (Moss-Racusin, 2014). Male leaders who take longer paternity leave than normal receive more negative evaluations than those who take shorter leave, and are seen as lower-status leaders compared to women who request leave (Gartzia et al., 2018). Men who hold traditionally feminine occupations tend to face more adverse backlash than women who do the same job (Eriksen and Einarsen, 2004; Heilman and Wallen, 2010; Moss-Racusin and Johnson, 2016). Men exhibiting typically feminine behavior such as advocating for others are judged as lacking in agency and competence (Bosak et al., 2018).

Looking beyond how counter-stereotypical individuals impact others’ perceptions, displaying cross-gendered roles also has an effect on the actors. Backlash and efforts to maintain cultural stereotypes (Rudman and Fairchild, 2004) motivate individuals who violate gender expectations to subsequently attempt to regain their self-esteem as a means of managing threats to self-worth. When someone behaves in contravention of traditional gender stereotypes—by successfully accomplishing a cross-gendered task, for instance—they might experience a fear of backlash (Iacoviello et al., 2021) and consequent social rejection, which negatively impacts the individual’s self-esteem. Thus, those who deviate from gender roles might be encouraged to avoid negative backlash and attempt to recover their status and self-esteem through a variety of strategies (Rudman et al., 2007), such as improving their gender conformity or hiding their cross-gendered behavior.

In sum, although counter-stereotypical behavior might contribute to the reduction of traditional gender norms, paradoxically, it might also serve to exacerbate gender inequalities.

## 2 The present research

The notion of gender identity threat suggests that when a person’s gender status is questioned, they will feel threatened and attempt to restore that status (e.g., Rudman et al., 2007; Rudman et al., 2012; Rudman and Fairchild, 2004). Thus, when individuals succeed in a cross-gendered field, they show higher levels of implicit self-esteem as a form of self-defense in response to an identity threat (Rudman et al., 2007). The aim of the present work is to delve further into this process by applying a more pertinent way of manipulating gender identity threat and by testing structurally crucial outcomes: attitudes related to maintaining the gender power imbalance, such as support for gender stereotypes and different types of sexism (Rudman et al., 2012).

In previous research, deviation from gender roles has been encouraged in the form of success in a cross-gendered domain (e.g., Heilman and Wallen, 2010; Rudman et al., 2007; Rudman and Fairchild, 2004). However, we argue that succeeding in traditionally opposite-gendered fields might not be a commonly experienced situation due to socio-psychological processes, such as the stereotype threat (Manzi et al., 2021; Spencer et al., 2016; Totonchi et al., 2021), the fear of being negatively evaluated (Villanueva-Moya and Expósito, 2021), or the fact that gender social norms are deeply internalized (Eagly, 1987; Ellemers, 2018). In the present study, we use a simpler and more ordinary situation that is more pertinent to the current social context in which deviating from gender roles in everyday tasks has become more normalized and legitimized. To elicit gender identity threat, participants were asked to choose between two similar products related to the traditional feminine stereotype or between two products related to the masculine stereotype, according to the experimental condition. We assume that this task is an inconsequential task that has no relevant implications for participants. Furthermore, we tested the impact of gender threat on outcomes related directly to the persistence and justification of gender inequality, such as support for gender stereotypes and sexism. Given that carrying out a counter-stereotypic task implies negative consequences, such as the experience of threat (see Rudman et al., 2007), a way to restore and defend the own self-esteem necessarily implies to support and confirm stereotypical attitudes that embrace traditional gender roles. We expect that male participants who are forced to choose a traditionally feminine product and female participants who must choose a typically masculine item will experience a gender identity threat that will lead to compensatory gender-system defense responses.

## 3 Materials and methods

### 3.1 Participants

We used a stratified random sample, representative of the adult population living in Chile’s five largest urban areas. Participants were 493 adults (*M*_age_ = 44.78, SD_age_ = 14.19, range = 18–69), 57% (*n* = 283) were women.

### 3.2 Procedure and design

Data were collected between September 2019 and March 2020 as part of a larger project conducted by a Measurement Center aimed at studying perceptions relating to a range of social issues. It was approved by a university Ethics Committee. All participants signed an informed consent form and engaged in computer-assisted personal interviews at their home addresses.

The study followed a 2×2 factorial design in which we manipulated gender identity threat and measured participants’ sex. To manipulate gender identity threat, participants were shown a single image with two similar daily life products and asked to choose one for themselves. The image also contained text indicating the type of product and a reference value. All items were from unknown brands and had the same reference value (USD 20). In the feminine condition (*n* = 242), participants were presented with two traditionally feminine products related to beauty: body creams. In the masculine condition (*n* = 251), participants were shown two traditionally masculine products related to manual skills: electric screwdrivers. Thus, we follow a 2 Gender identity threat (masculine vs. feminine) x 2 Sex (men vs. women) design, and participants were randomly assigned to one of these threat conditions. According to this design, threat to gender identity was expected to emerge for men in the feminine condition and for women in the masculine condition. Following exposure, participants filled out scales for the dependent variables.

### 3.3 Measurements

#### 3.3.1 Sexism

We used eight items from the Ambivalent Sexism Inventory (Glick and Fiske, 1996), adapted to the Chilean population by Cárdenas et al. (2010). The items included both hostile (e.g., “Many women are actually seeking special treatment under the guise of equality”) and benevolent (e.g., “Women, as compared to men, tend to have a more refined sense of culture and good taste”) dimensions of the scale. Participants indicated their agreement with each item using a scale ranging from 1 (Strongly disagree) to 5 (Strongly agree). Scores of all items were averaged; a higher score on the scale indicates higher levels of sexism. Cronbach’s alpha for this scale was acceptable (*α* = 0.79).

#### 3.3.2 Belief in sexism shift

We used four items to measure a novel form of sexism: the idea that men—rather than women—are the current victims of sexism (Zehnter et al., 2021). Items were created for the present study based on previous literature that shows that social progress for women implies losses for men (Ruthig et al., 2017). Cronbach’s alpha for this scale was acceptable (*α* = 0.78).

#### 3.3.3 Traditional gender stereotypes

To measure masculine and feminine stereotypes, participants indicated the extent to which seven traits were characteristic of both men and women. Four traits were traditionally masculine (ambition, superior intelligence, self-confidence, and independence) and three traits were traditionally assigned to women (kindness, cooperation, and being a good listener). Participants indicated how characteristic each trait was of men or women using a scale ranging from 1 (Very uncharacteristic) to 5 (Highly characteristic). Evidence of the validity of this scale was obtained in another study (see Jiménez-Moya et al., 2022a). Scores of all items of each sub-scale were averaged; a higher score indicates higher levels of gender stereotypes. Cronbach’s alpha was acceptable for masculine (*α* = 0.75) and feminine (*α* = 0.80) stereotypes.

### 3.4 Data analysis

Data analyses were conducted using R version 4.5.0. Four 2 Gender identity threat (masculine vs. feminine) x 2 Sex (men vs. women) ANOVAs were conducted using sexism, belief in sexism shift, masculine stereotypes, and feminine stereotypes as dependent variables. When interactions were significant, simple effects analyses were conducted.

## 4 Results

Descriptive statistics and bivariate correlations are shown in Table 1.

**Table 1:** Means, standard deviations, and correlations with confidence intervals.

Variable	M	SD	1	2	3
1. Sexism	2.84	0.68			
2. Belief in sexism shift	2.72	0.76	0.58** [0.52, 0.64]		
3. Masculine Stereotypes	3.64	0.69	0.11* [0.02, 0.20]	0.11* [0.02, 0.20]	
4. Feminine Stereotypes	4.05	0.63	0.06 [−0.03, 0.15]	0.04 [−0.05, 0.13]	0.38** [0.31, 0.46]

M and SD are used to represent mean and standard deviation, respectively. Values in square brackets indicate the 95% confidence interval for each correlation. * indicates *p* < 0.05. ** indicates *p* < 0.01.

### 4.1 Sexism

**Figure 1:**
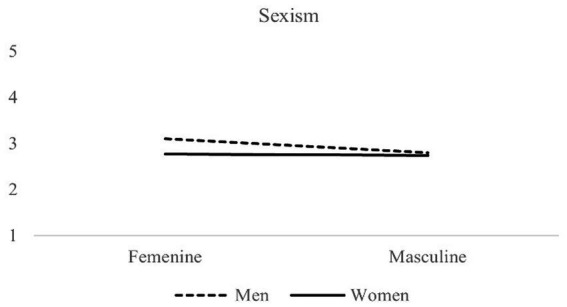
Sexism levels on the part of male and female participants in the feminine and masculine gender identity threat conditions.

A significant Gender identity threat x Sex interaction was found, *F*(1,489) = 5.30, *p* = 0.022 (see Figure 1). Simple effects analysis revealed that in the feminine condition, men exhibited higher levels of sexism compared with women: *F*(1,489) = 15.47, *p* = 0.0001. Also, men expressed higher levels of sexism in the feminine condition compared to the masculine condition: *F*(1,489) = 10.98, *p* = 0.001. In other words, and in line with our hypothesis, when male participants were asked to choose a body cream for themselves, they subsequently expressed higher levels of sexism compared to women in the same condition, and also compared to men who had to choose an electric screwdriver.

No differences were observed in the masculine condition between women and men—*F*(1,489) = 0.46, *p* = 0.499—or between conditions in women: *F*(1,489) = 0.101, *p* = 0.750. In other words, women did not express higher levels of sexism when they were asked to choose a traditionally masculine product.

### 4.2 Belief in sexism shift

We found a main effect of the gender identity threat condition. Participants in the feminine condition presented higher levels of belief in sexism shift compared with the masculine condition, *F*(1,489) = 8.33, *p* = 0.004. That is, when people had to choose a body cream for themselves, they tended to adhere more to the idea that today, men are more discriminated against compared to women.

The Gender identity threat x Sex interaction was not significant—*F*(1,489) = 0.06, *p* = 0.813; nor was the main effect of sex, *F*(1,489) = 3.01, *p* = 0.083.

### 4.3 Masculine stereotypes

We found a main effect of sex. Men expressed higher levels of masculine stereotypes compared with women, *F*(1,489) = 10.21, *p* = 0.001. This means that, overall, men adhere more strongly to traditional stereotypes about masculinity. The Gender identity threat x Sex interaction was not significant—*F*(1,489) = 0.06, *p* = 0.805—nor was the main effect of gender identity threat condition, *F*(1,489) = 0.38, *p* = 0.540.

### 4.4 Feminine stereotypes

**Figure 2:**
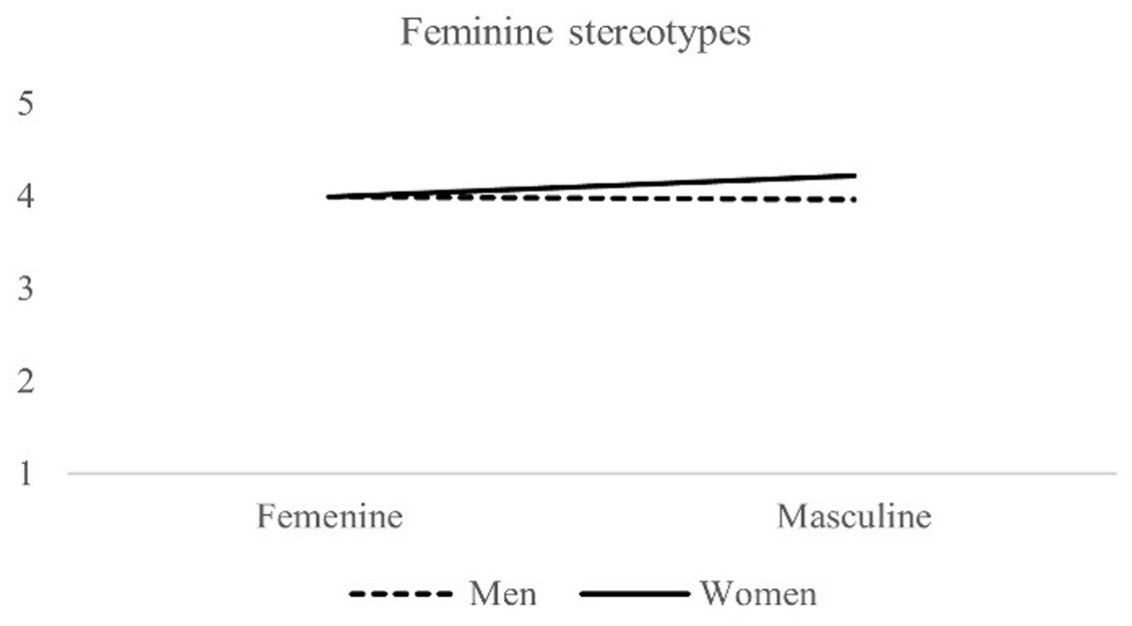
Feminine stereotype levels exhibited by male and female participants in the feminine and masculine gender identity threat conditions.

Results showed a significant Gender identity threat x Sex interaction, *F*(1,489) = 5.09, *p* = 0.025 (see Figure 2). Simple effects analysis revealed that in the masculine condition, women presented higher levels of feminine stereotypes than men, *F*(1,489) = 9.91, *p* = 0.002. Furthermore, women showed higher levels of feminine stereotypes in the masculine condition compared to the feminine condition: *F*(1,489) = 9.72, *p* = 0.002. In other words, according to our hypothesis, women who were asked to choose an electric screwdriver expressed greater endorsement of the traditional feminine stereotype compared to men in the same condition, and also compared to women who had to choose a body cream for themselves. No differences were observed in the feminine condition between women and men, *F*(1,489) = 0.002, *p* = 0.968, or between conditions in men, *F*(1,489) = 0.08, *p* = 0.772.

## 5 Discussion

Gender roles are fixed social norms that establish how men and women should behave (Eagly and Karau, 2002; Eagly and Wood, 2012) and imply negative consequences for both men and women (Gartzia et al., 2018; Schmader, 2023) across their time course. Counter-stereotypical models emerge as a way of mitigating the notion and practice of traditional gender roles (Chhaochharia et al., 2022; Olsson and Martiny, 2018). However, holding a successful counter-stereotypical role might also trigger a gender identity threat which, paradoxically, might lead to conformity with gender roles or to hiding cross-gendered behaviors (Heilman and Wallen, 2010; Rudman et al., 2012; Rudman and Fairchild, 2004). The aim of the present work was to analyze whether a simple task that might take place on a daily basis—choosing a counter-stereotypical product for oneself—might provoke a gender identity threat and, in turn, magnify attitudes that justify and perpetuate gender inequality. Results confirmed our hypothesis: men who experienced a gender identity threat—by having to choose a body cream for themselves—subsequently exhibited higher levels of sexism compared to men that were not threatened: men asked to choose a traditionally masculine product. Furthermore, women who were threatened in their gender identity by being forced to choose an electric screwdriver subsequently demonstrated greater endorsement of the positive traditional feminine stereotype compared to women who did not experience a gender identity threat.

Our results demonstrate that even a minor routine consumer decision can be sufficient to elicit compensatory gender-system defense responses. The way to face self-esteem threats elicited by counter-stereotypic tasks, consists in adopting attitudes that support traditional gender views. Merely choosing a body-care item (for men) or a manual tool (for women) shifted subsequent endorsement of attitudes that justify and perpetuate gender inequality, suggesting that gender identity is chronically monitored and reactively protected in mundane contexts. The asymmetrical pattern is informative and coherent with each group status: men exposed to a feminine choice demonstrated increased sexism—a status-protective response consistent with the higher structural position of the male category (Iyer, 2022; Rios et al., 2018; Tajfel and Turner, 1979); whereas women exposed to a masculine choice selectively increased endorsement of positive feminine traits—a strategy that safeguards valued in-group distinctiveness without directly challenging gender inequality (Jimenez-Moya et al., 2012; Tajfel and Turner, 1979; Van Bezouw et al., 2021). Men’s increased sexism following a feminine cue aligns with status-incongruity and precarious manhood claims: when masculinity is questioned, men preferentially deploy hierarchy-affirming beliefs to restore status (see Dahl et al., 2015). Women’s reinforcement of positive feminine traits maps onto social-creativity strategies within social identity theory: when direct status challenge is costly or risky, revaluing ingroup-typical attributes protects identity with minimal backlash (Tajfel and Turner, 1979). Notably, belief in a “sexism shift” (men as current victims) also moved upward under a feminine cue, consistent with zero-sum threat narratives that legitimize status protection. An intersectional lens suggests that these pathways may vary by class, age, or ideology—dimensions that confer different costs and affordances for overt vs. covert defense. Together, the results depict hierarchy-consistent restoration: higher-status group members lean on system-justifying ideology (sexism), whereas lower-status members gravitate to identity-affirming positivity that preserves belonging while avoiding sanctions. In sum, both men and women experienced a gender identity threat when they chose for themselves a counter-stereotypical product (see Rudman et al., 2007; Rudman et al., 2012; Rudman and Fairchild, 2004), and showed backlash aimed at justifying and perpetuating gender inequality, such as sexism and traditional gender stereotypes. The implications of these findings are crucial. Encouraging counter-stereotypical roles seems to, paradoxically, trigger the persistence of gender inequality if a gender identity threat is activated. However, to achieve gender equality it is crucial that men and women embrace counter-stereotypic roles (e.g., Buckley et al., 2022; Chhaochharia et al., 2022). In this complex context, we argue that the long-term aim should be to abolish traditional gender norms that connect specific products, tasks or roles with men and women. This way using certain products would not elicit gender identity threats. In short, the notion of gender identity threat and its effects, needs to be incorporated into interventions aimed at reducing support for traditional gender roles (e.g., Olsson and Martiny, 2018; Olivetti et al., 2020) in order to be more effective, successful, and suitable for achieving a real change in terms of gender equality. This is particularly consequential, as interventions with this goal are needed to reduce support for gender stereotypes (Eagly and Koenig, 2021; Koenig and Eagly, 2014).

Our results also showed a positive ingroup bias among male participants, who expressed greater support for the traditional masculine stereotype. This result is coherent with ingroup bias literature, as the masculine traits we used could be evaluated as positive and desirable (Brewer, 1999; Verkuyten, 2021). Results also showed a main effect of the feminine condition on the belief in sexism shift, as participants who had to choose a body cream for themselves tended to agree with the idea that today men are more discriminated against than women. The feminine condition might be perceived as a saliency of feminine issues in general, which might trigger some threatening feelings. This result is in line with previous research showing that people, particularly men, tend to engage in behaviors that restore their status and gender identity after their gender identity has been threatened (Dahl et al., 2015; Fowler and Geers, 2017; Vescio et al., 2021; Weaver et al., 2013).

It is relevant to discuss why some outcomes move in the hypothesized direction, but others did not. Identity threats often elicit domain-congruent defenses. For men, endorsing sexist beliefs is a direct, hierarchy-maintaining strategy that restores a threatened high-status identity; for women, affirming positive feminine stereotypes is a lower-conflict, identity-sustaining route that preserves valued distinctiveness without openly challenging the hierarchy Iyer, 2022; Tajfel and Turner, 1979. In contrast, broad gender-trait stereotypes (especially masculine traits) showed a stable ingroup-favoring baseline among men that did not shift with a single exposure, consistent with their more trait-like character. Finally, “belief in sexism shift” captured a general zero-sum ideology that was modestly heightened by the feminine cue but not differentially by participant gender, suggesting a context-wide salience effect rather than a tightly targeted, sex-specific defense. Together, we argue that these patterns indicate that brief, everyday counter-stereotypical cues channel defenses into status-restoring (men into sexism) vs. identity-affirming (women into positive feminine traits) pathways, while global or trait-level judgments are less labile in the moment.

Limitations to this study also allow us to suggest directions for future research. First, our manipulation contrasted specific product categories (body cream vs. electric screwdriver) that may differ not only in terms of gender connotation but also in terms of perceived utility, familiarity, or desirability. Employing multiple, pretested stimulus sets and including a gender-neutral control condition would sharpen causal inferences. Second, data were collected in Chile between September 2019 and March 2020, a period marked by particularly salient public debates over feminism and social change. Such heightened salience likely increased baseline identity vigilance and lowered thresholds for threat appraisals. For men, a feminine cue may have more readily activated status-protective defenses (higher sexism). For women, the same climate could have made ingroup-affirming responses (endorsing positive feminine traits) more available and normatively safe. These context effects caution against direct generalization to settings with lower gender-politics salience. We therefore frame our findings as evidence for threat-response mechanisms observable in mundane choices, while underscoring that their magnitude may vary with cultural moment and public discourse; cross-cultural and longitudinal replications are warranted. Third, samples from different cultural and geographical backgrounds would help to support the cross-cultural validity of our findings. Finally, specific attitudes related to the approval of traditional gender norms might moderate these findings (Borinca et al., 2021; Falomir-Pichastor et al., 2019).

Taken together, our results show that even trivial, everyday deviations from prescribed gendered norms can trigger system defense responses that reaffirm and justify gender inequality. The restoration pathways were differentiated: men responded to a feminine cue with more pronounced sexism, whereas women responded to a masculine cue by reaffirming positive feminine traits—patterns consistent with status and identity-protective motives. Interventions that rely on counter-stereotypical exposure must therefore also buffer gender identity threat and anticipated backlash, which might contribute to the perpetuation of gender inequality.

## Data Availability

The raw data supporting the conclusions of this article will be made available by the authors, without undue reservation.
